# Atypical hemispheric re-organization of the reading network in high-functioning adults with dyslexia: Evidence from representational similarity analysis

**DOI:** 10.1162/imag_a_00070

**Published:** 2024-01-22

**Authors:** Eddy Cavalli, Valérie Chanoine, Yufei Tan, Jean-Luc Anton, Bruno L. Giordano, Felipe Pegado, Johannes C. Ziegler

**Affiliations:** Lyon 2 Univ, EMC, Lyon, France; Aix Marseille Univ, CNRS, LPL, Aix-en-Provence, France; Aix-Marseille Univ, ILCB, Aix-en-Provence, France; Aix Marseille Univ, CNRS, LPC, Marseille, France; Aix Marseille Univ, CNRS, INT, Marseille, France; Université Paris Cité, CNRS, LaPsyDé, Paris, France

**Keywords:** high-functioning adults with dyslexia, representational similarity analysis (RSA), semantic similarity, orthographic similarity, fMRI

## Abstract

It has been argued that university students with dyslexia compensate for their reading deficits by a neural re-organization of the typical reading network, where the lexical representations of words are (re-)structured according to semantic rather than orthographic information. To investigate the re-organization of neural word representations more directly, we used multivariate representational similarity analyses (RSA) to find out which brain regions of the reading network respond to orthographic and semantic similarity between 544 pairs of words and whether there were any differences between typical and dyslexic readers. In accordance with the re-organization hypothesis, we predicted greater similarity (i.e., correlation of neural dissimilarity matrices) in adult dyslexic than in typical readers in regions associated with semantic processing and weaker similarity in regions associated with orthographic processing. Our results did not confirm these predictions. First, we found sensitivity to semantic similarity in all three subparts of the fusiform gyrus (FG1, FG2, and FG3) bilaterally. Adults with dyslexia showed less (rather than more) sensitivity to semantic similarity in the posterior subpart of fusiform gyrus (FG1) in the left hemisphere. Second, in typical readers, sensitivity to orthographic information was not only found in the left fusiform gyrus (FG1, FG2, and FG3) but also in left inferior frontal gyrus (IFG). Adults with dyslexia, in contrast, did not show sensitivity to orthographic information in left IFG. However, they showed increased sensitivity to orthographic information in the right hemisphere FG1. Together, the results show abnormal orthographic processing in left IFG and right FG1 and reduced semantic information in left FG1. While we found evidence for compensatory re-organization in adult dyslexia, the present results do not support the hypothesis according to which adults with dyslexia rely more heavily on semantic information. Instead, they revealed atypical hemispheric organization of the reading network that is not restricted to the typical left language hemisphere.

## 1 Introduction

Developmental dyslexia (DD) is a severe, specific, and long-lasting neurodevelopmental reading disorder that prevents children from becoming efficient and fluent readers despite normal intelligence and appropriate educational opportunities (for reviews, see Démonet et al., 2004; Norton et al., 2014; Shaywitz & Shaywitz, 2005). Although deficient phonological and language development are among the major causal factors of DD (Hulme et al., 2015), the proximal core deficit lies in poor decoding and visual word recognition skills, which seem to prevent the automatization of these processes through self-teaching (Perry et al., 2019; Ziegler et al., 2020).

In the present study, we were interested in university students who were diagnosed with DD when they were children^1^. This is a rather special group of people because it is not easy to get through the school curriculum and engage in higher education when reading is effortful and slow (Beddington et al., 2008). Indeed, most of the children with dyslexia tend to find occupations that minimize reading activities (Guthrie & Wigfield, 1999; Morgan et al., 2008). Thus, university students with dyslexia must have found a way to compensate. Although they typically remain slow readers and poor spellers (Cavalli et al., 2016; Swanson, 2012), their text comprehension seems to be relatively spared (Cavalli et al., 2019; Deacon et al., 2012). In the present article, we were interested in finding out whether there was any evidence for compensatory re-organization of the reading network in adults with dyslexia that allows these students to compensate for their impoverished word recognition skills.

It has been suggested that one of the compensatory mechanisms that allows students with dyslexia to cope with orthographic processing deficits is the reliance on contextual information and semantics (Cavalli et al., 2016; Snowling, 2000; Stanovich, 1980). For example, Martin et al. (2013) showed that morpho-semantic knowledge is relatively preserved in university students with dyslexia, whereas phonological processing is clearly impaired (see also Law et al., 2015). Specifically, in the same population, Cavalli, Duncan, et al. (2017) found a dissociation between intact morpho-semantic abilities and impaired phonological processing and the magnitude of this dissociation correlated with the students’ reading level.

Such behavioral evidence could be taken to suggest that university students with dyslexia develop compensatory mechanisms. As a consequence, they might show a neural re-organization of the reading network, in which semantic representations could be activated more strongly and possibly faster than orthographic representations during reading (for evidence in MEG, see Cavalli, Colé et al., 2017). Consistent with this idea, some fMRI studies indeed reported an overactivation of frontal areas during tasks of word and pseudoword reading that could potentially be associated with semantic processing (Brunswick et al., 1999; Salmelin et al., 1996; Shaywitz et al., 1998). However, the overactivation in frontal areas could be due to articulatory compensation or increased effort (Hancock et al., 2017; Richlan et al., 2011; Richlan et al., 2009). Moreover, the few neuroimaging studies that specifically investigated semantic processing in dyslexia sentence reading (e.g., manipulating the semantic appropriateness of a the final word in a sentence, Helenius et al. 1999), or a pseudohomophone reading task (Paz-Alonso et al., 2018), or a semantic judgment task (Rüsseler et al., 2007), typically found weaker (rather than stronger) activation in the left middle and superior temporal cortex, delayed N400, or reduced hippocampal activation in dyslexics than controls.

With respect to the question of interest (i.e., is there any evidence for compensatory re-organization of the reading network in adult with dyslexia?), one elegant way to investigate restructuring of lexical representations is to use representational similarity analysis (RSA, Kriegeskorte et al., 2008), which is based on the idea that words (or other representations), which are similar on a given dimension (e.g., semantics), should produce similar patterns of neural responses across voxels in a region that “cares” about that particular dimension. For example, in a semantic processing region, *shirt* and *dress* should produce more similar neural responses than *shirt* and *book.* In turn, in an orthographic processing region, *shirt* and *ship* should produce more similar neural responses than *shirt* and *book*.

**Fig. 1.**
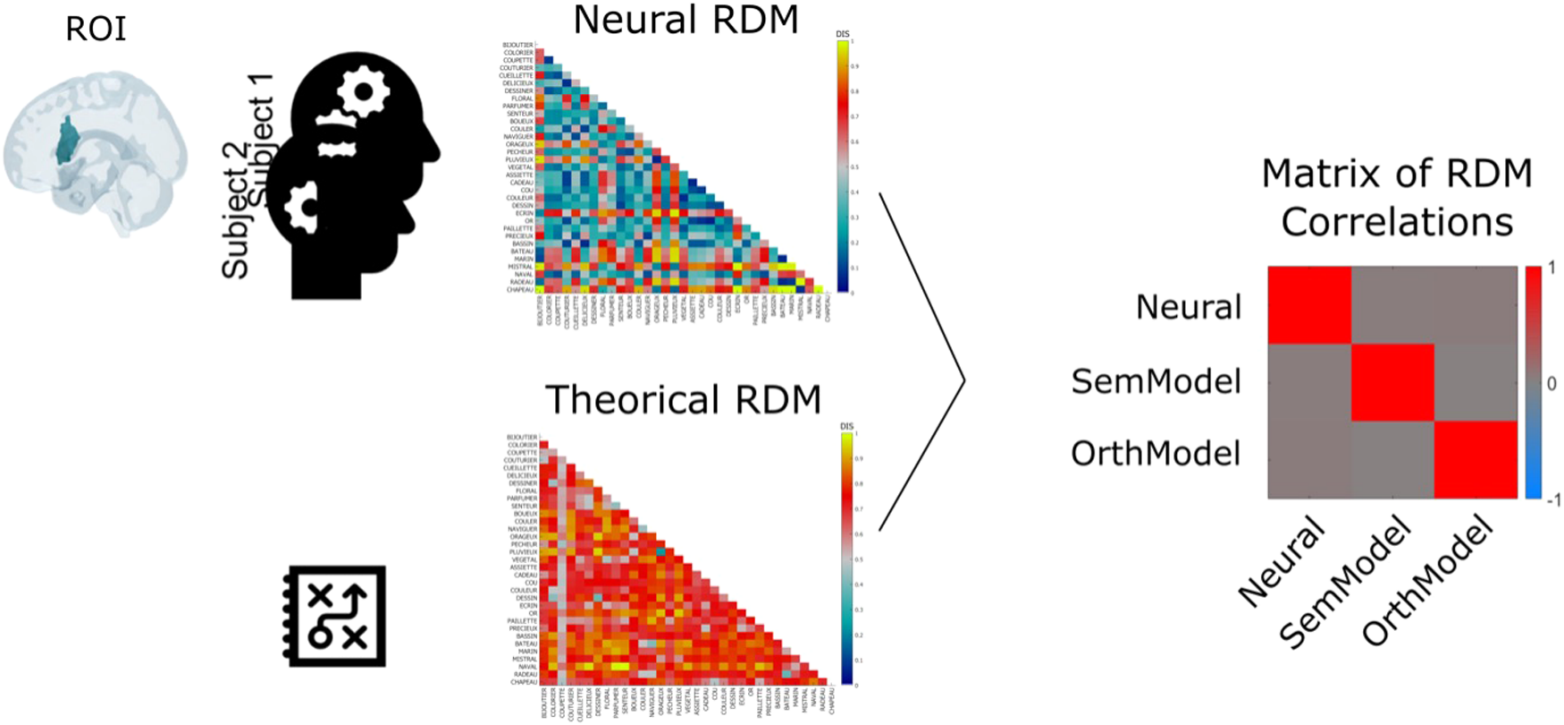
Principle of representational similarity analysis (RSA) as implemented in the present study. Neural responses to single words are measured in a given ROI for each participant. The neural dissimilarity matrix (neural RDM) between all word pairs is then compared to a theoretically relevant RDM (theoretical RDM) using a second-order correlation (i.e., matrix of RDM correlations)

The logic of the present study is illustrated in Figure 1. Participants were presented with individual words and the neural responses to these words were measured in different regions of interest (ROIs). This makes it possible to establish a neural representational similarity (or inversely, the dissimilarity) matrix between all word pairs (i.e., neural RDM). The neural dissimilarity matrices were then being compared to theoretically relevant (dis)similarity matrices. In our case, we used a semantic (dis)similarity and an orthographic (dis)similarity matrix. We then computed second-order correlations to find out to what extent the neural RDMs were similar to the theoretical RDMs in a given ROI. A significant second-order correlation between the neural RDM and the theoretical RDM means that a given ROI is sensitive to the information that is captured in the theoretical (dis)similarity matrix. If this logic is applied to our original research question (i.e., is there any evidence for compensatory re-organization of the reading network in adult dyslexia?), we would predict greater second-order correlations in adult dyslexics than in controls for the semantic dimension and weaker second-order correlations for the orthographic dimension, both in ROIs associated with either semantic or orthographic processing. We acknowledge that one could have increased reliance on higher level semantic or even contextual information using a sentence reading task. However, in the present article, we were not interested in investigating reading strategies, as semantic or contextual predictions, but neural re-organization of lexical representations that are part of the reading network. In that respect, reading aloud of isolated words seemed like a good task because it requires a precise response on every trial and it is well known that reading aloud activates lexical representations because lexicality and frequency effects are extremely robust in this task (see Coltheart et al., 2001).

**Fig. 2.**
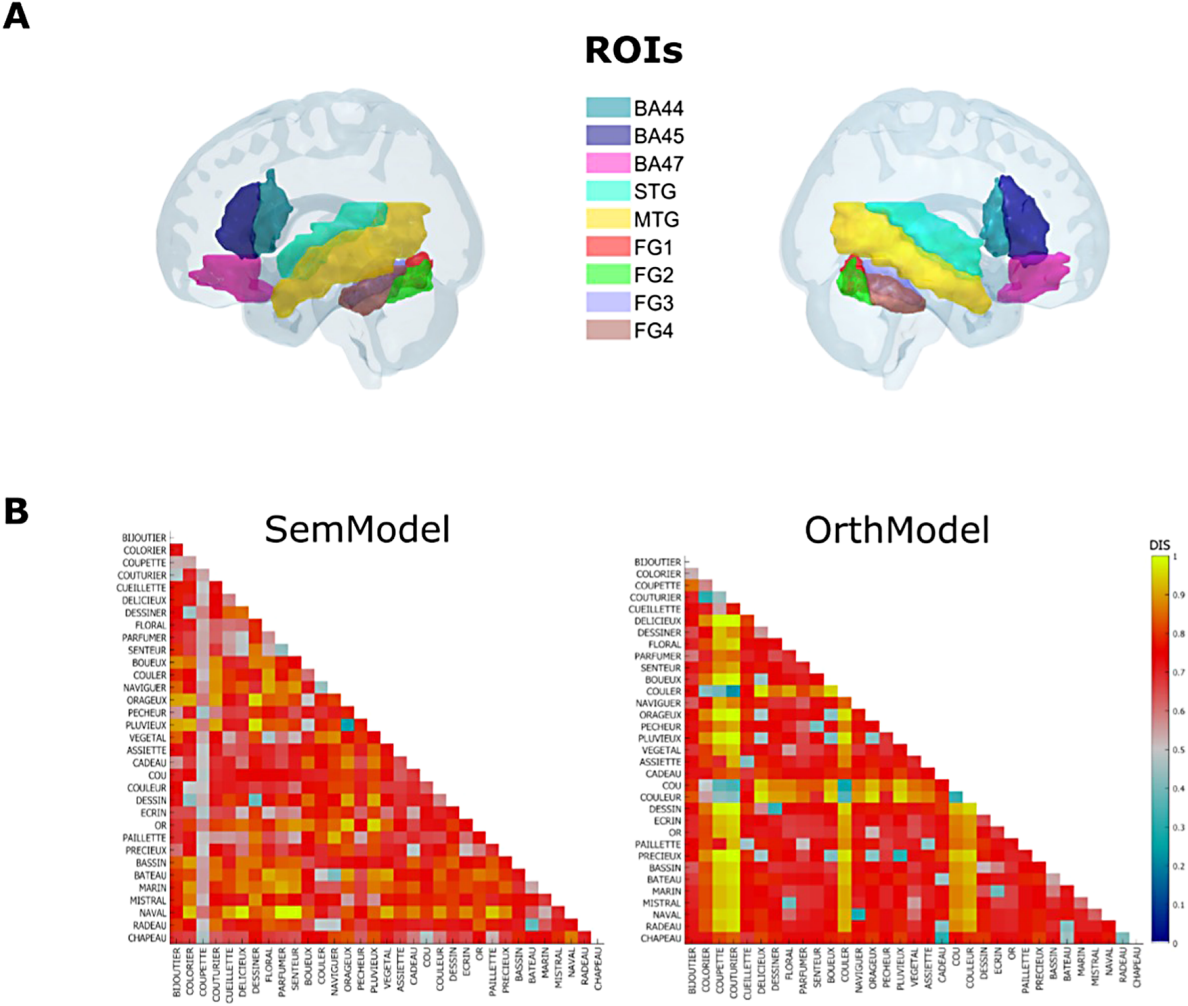
(A) Anatomical ROIs used in the RSA and projected on a cortical surface (left and right view of an MNI brain mesh). They were extracted from the SPM Anatomy toolbox (Eickhoff et al., 2005) and WFU PickAtlas Standard Atlases (Maldjian et al., 2003): three subparts of inferior frontal gyrus (Brodmann areas BA44, BA45, and BA47), one region of superior temporal gyrus (STG), one region of middle temporal gyrus (MTG), and four subparts of the fusiform gyrus (FG1, FG2, FG3, and FG4) were thus created (see section “Region of interests” for more details). (B) The two theoretical matrices used in our RSA: a semantic (dis)similarity matrix (SemModel) and an orthographic (dis)similarity matrix (OrthModel).

As illustrated in Figure 2A, we applied RSA, a type of multi-voxel pattern analysis (MVPA) (Fischer-Baum et al., 2017) using nine anatomical ROIs that map the left-hemisphere reading network along with their right-hemisphere homologues (see the “Regions of Interest” method section for more details). The ROIs included bilateral inferior frontal gyrus (IFG), superior and middle temporal gyrus (STG and MTG), and fusiform gyrus (FG). Previous research has shown that both left IFG and left FG are not uniform region, neither anatomically nor functionally. Indeed, it has been shown that left IFG subdivisions responded to different linguistic processes, usually involving posterior-dorsal left IFG (BA44) in phonological and syntactic processing (Hagoort, 2005; Hagoort & Indefrey, 2014) and anterior-inferior left IFG (BA45 and BA47) in semantic processing (Friederici, 2012). Similarly, the different left FG subdivisions respond to different visual word-recognition processes, exhibiting a posterior-to-anterior gradient that becomes increasingly sensitive to high-level orthographic features along this axis (Lerma-Usabiaga et al., 2018; Vinckier et al., 2007; Zhan et al., 2023; Zhao et al., 2017). Therefore, these ROIs were subdivided according to the Julich-Brain Atlas (Amunts et al., 2020), a 3D atlas aligned with the MNI-Colin27 space and defined by probabilistic cytoarchitectonic maps linked to functional data, resulting in three subparts of bilateral IFG (BA44, BA45, and BA47) and four posterior-to-anterior subparts of bilateral FG (FG1, FG2, FG3, and FG4).

To find out what kind of information (orthographic or semantic) is represented in each ROI, we computed two theoretical matrices, a semantic (dis)similarity matrix (SemModel) and an orthographic (dis)similarity matrix (OrthModel). For the semantic matrix, we used a vector space distributed semantic model (van der Maaten, 2014), which was trained on a large French corpus (Frwiki, 11 GB, 914,601,321 tokens). For the orthographic matrix, we used a weighted Levenshtein distance, which gave extra weight to initial and final orthographic overlap (Grainger et al., 2016). The two matrices are presented in Figure 2B.

In sum, in the present study, we asked university students with and without dyslexia to read aloud single words several times to obtain robust neural responses to individual words in fMRI. We then used a multivariate RSA analysis to find out what regions of the reading network processed orthographic and semantic information. Finally, we compared the groups to find out whether university adults with dyslexia would present a difference in their orthographic and semantic representations relative to typically developing university adults, as recently suggested by Keshavarzi et al. (2022).

## 2 Methods

### 2.1 Participants

Twenty adults with dyslexia (11 women and 9 men) and 22 typical adult readers (12 women and 10 men), all of whom were university students and monolingual native French speakers, were recruited at Aix-Marseille University (France). The two groups were matched on both chronological age (*t*(40) = 0.46, *p* = 0.78) and educational level (*t*(40) = 0.21, *p* = 0.82). The two groups were also matched on academic program with approximately 60% of the participants were enrolled in Social and Humanities and around 40% in Natural Sciences. None of the participants had any known neurological and/or psychiatric disorders and all reported normal or corrected-to-normal hearing and vision. The experiment was conducted in accordance with the Declaration of Helsinki and with the understanding and written consent of all participants. The experiment was granted ethical approval by a national review board (Committee for the Protection of Individuals, CPP 2017-A03614–49).

University students with dyslexia were recruited following a diagnosis of dyslexia established by a regional reference center for the diagnosis of learning disabilities (*Centre de Référence des Troubles des Apprentissages*) at the *Hôpital Salvator* in *Marseille* [Center for the diagnosis of learning disabilities, Salvator Hospital], and/or by a specialized disability support service (*Mission Handicap)* of Aix-Marseille University medical service. They had all received a formal diagnosis of dyslexia during primary school and had received remedial teaching for an average of 5.34 years (SD = 0.41). Moreover, they reported having experienced major difficulties in learning to read in childhood and adolescence. These difficulties were confirmed prior to inclusion in the present study using the French version of the Adult Reading History Questionnaire-Revised (ARHQ-R, Lefly & Pennington, 2000), a self-report questionnaire for which all participants with dyslexia had to have score above the cutoff score of 0.43 (Bjornsdottir et al., 2013). This questionnaire is widely used to screen for dyslexia in adults (e.g., for English see Deacon et al., 2012; for French see Marchetti et al., 2023). It consists of 23 Likert-scale items, including questions on reading habits, reading and spelling abilities, reading speed, attitudes toward school and reading, additional assistance received, repeating grades or courses and effort required to succeed in elementary school, secondary school, post-secondary education, and current life.

All participants were administered a set of neuropsychological tasks (see Table 1 for detailed results), including tasks to estimate both nonverbal IQ (by using the Raven’s matrices, Raven & Raven 2003) and verbal IQ (by using a standardized vocabulary task, the French version of the vocabulary EVIP scale; Dunn et al., 1993; see Cavalli et al., 2016). All participants performed above the fifth percentile on both nonverbal and verbal IQ tasks, thereby confirming that none of the participants presented a deficit in nonverbal reasoning and in semantic oral language skills. Moreover, potential participants with a formal diagnosis of specific language impairment or other impairments that could impact language ability (e.g., autism spectrum disorder) were not included in the present study. The neuropsychological assessment also included reading and reading-related tasks assessing skills known to be persistently impaired in adults with dyslexia and even for those who successfully manage to study at the university level (Brèthes et al., 2022; Cavalli, Colé et al., 2017; Martin et al., 2010). These tests included reading fluency (measured by the Alouette test, which is considered a “gold standard” in France for the assessment of dyslexia in children and adolescents but also in adults; see Cavalli, Colé et al., 2017) and phonological processing and decoding skills (measured by pseudoword reading, phonemic awareness, and phonological short-term memory (STM)). These tests were taken from EVALEC, a computerized battery for the assessment of reading and reading-related skills (see Sprenger-Charolles et al., 2005, and Cavalli et al., 2016; Cavalli, Colé et al., 2017, for reliability measures on these tasks).

**Table 1.** Participants’ characteristics and means (standard deviations) on the cognitive and language assessment tests for both dyslexics and typical readers.

	Dyslexic readers (N = 20)	*p*-Value	Typical readers (N = 22)	Cohen’s *d*
Chronological age	22.7 (4.2)	0.80	23.1 (3.7)	-
Educational level	2.9 (1.3)	0.75	3.3 (1.2)	-
Reading fluency^a^	368.7 (72.9)	***	491.4 (59.8)	-1.8
ARHQ-R^b^	0.58 (0.08)	***	0.32 (0.08)	-3.3
Vocabulary (verbal IQ)	38.2 (5.1)	0.51	39.1 (4.6)	-
Non-verbal IQ	41.6 (8.3)	0.79	42.2 (7.1)	-
Pseudoword reading (efficiency^c^)	0.7 (0.2)	***	1.3 (0.3)	-2.3
Phonemic awareness (efficiency^c^)	29.4 (9.0)	**	53.9 (17.1)	-1.6
Phonological STM (efficiency^c^)	0.7 (0.2)	**	1.1 (0.2)	-2.0

Effect sizes (Cohen’s *d*) for group comparison are also presented.

a Alouette Standardized Reading Test (Cavalli, Colé et al., 2017).

b Adult Reading History Questionnaire-Revised (Lefly & Pennington, 2000).

c Efficiency score = (accuracy/response time) * 10.

**p < 0.01, and ***p < 0.001.

The participants’ characteristics and group comparisons on neuropsychological tasks are presented in Table 1. As expected, the results on the standardized reading fluency test showed that the score of adults with dyslexia was significantly lower than that of the controls (18 out of 20 dyslexics were below the 1sd cutoff score of 432; *t*(40) = -5.98; *p* < 0.001; Cohen’s *d* = -1.8). The results on the ARHQ-R showed that adults with dyslexia obtained significantly higher scores (i.e., more impaired) than the controls (19 out of 20 dyslexics were above the 1sd cutoff score of 0.40; *t*(40) = 10.1; *p* < 0.001; Cohen’s *d* = -3.3). In addition, adults with dyslexia displayed significant lower pseudoword reading efficiency scores (19 out of 20 dyslexics were 1sd below the cutoff score of 1.0), as well as lower phonemic awareness score (17 out of 20 dyslexics were below1sd below the cutoff score of 36.8) and phonological STM efficiency scores (15 out of 20 were 1sd below the cutoff score of 0.9) compared to the control group (all *p* < 0.001; all Cohen’s *d*s above -1.6). In contrast, the two groups did not differ significantly on nonverbal IQ score (*t*(40) = -0.26; *p* = 0.79), nor on vocabulary (*t*(40) = -0.65; *p* = 0.51).

### 2.2 Stimuli

**Fig. 3.**
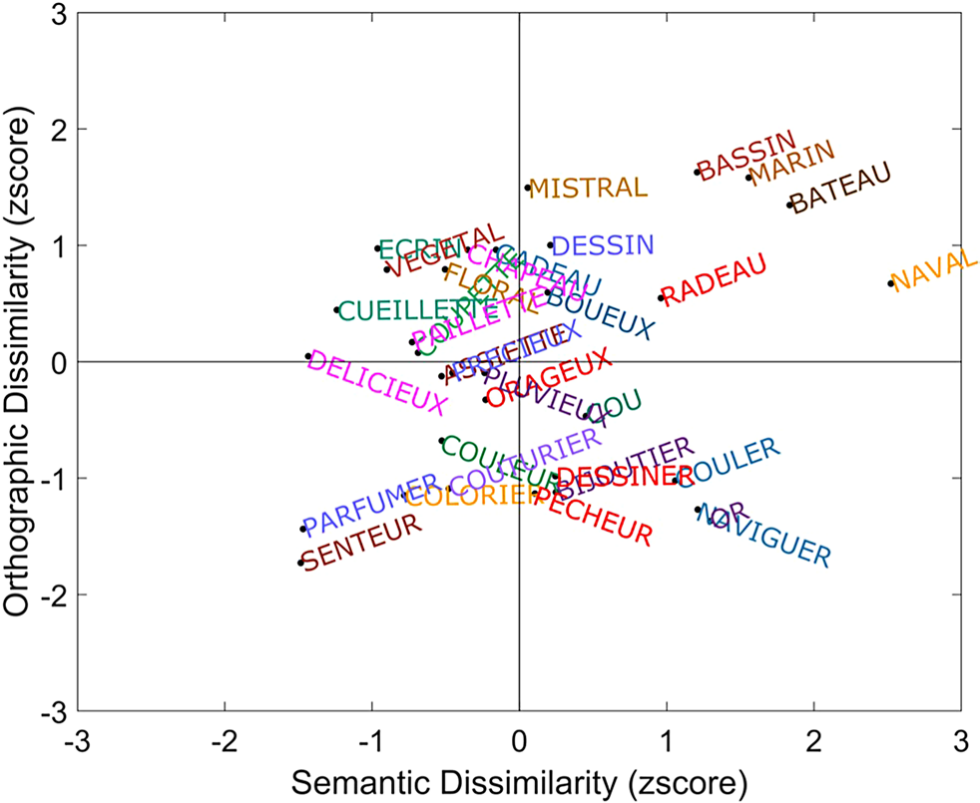
Multidimensional scaling (MDS) of our 33 words in two dimensions, which reflect orthographic distance (using Levenshtein distance) and semantic distance (using stochastic neighbor embeddings). The distance between a pair of words represents the similarity of them. The farther away two words are, the more dissimilar they are to each other.

We selected 33 words that belonged to two distinct semantic categories (“art” and “water”) and that were either orthographically similar or dissimilar to some of the other words. Word frequencies ranged from 1 to 125.8 per million (Mean = 23.79, SD = 36.65) (New et al., 2004), and word lengths ranged from 2 to 10 letters (Mean = 6.85, SD = 1.70). Words were chosen with the constraint that the orthographic similarity matrix and the semantic similarity matrix were not correlated. To do so, we conducted a multidimensional scaling (MDS) analysis to show that the selected words indeed occupied all quadrants of the orthographic-semantic distance space (see Fig. 3). Besides being a member of the same semantic category or not, the semantic distance was calculated for each pair of words based on a distributional semantic model, a recent version of stochastic neighbor embeddings (SNE; Hinton & Roweis, 2002) using two tree-based algorithms (van der Maaten, 2014). The orthographic distance was equal to the minimum number of characters that must be deleted, inserted, or replaced to move from one string to another (Levenshtein distance) with an extra weight given to initial and final overlap.

### 2.3 Tasks

**Fig. 4.**
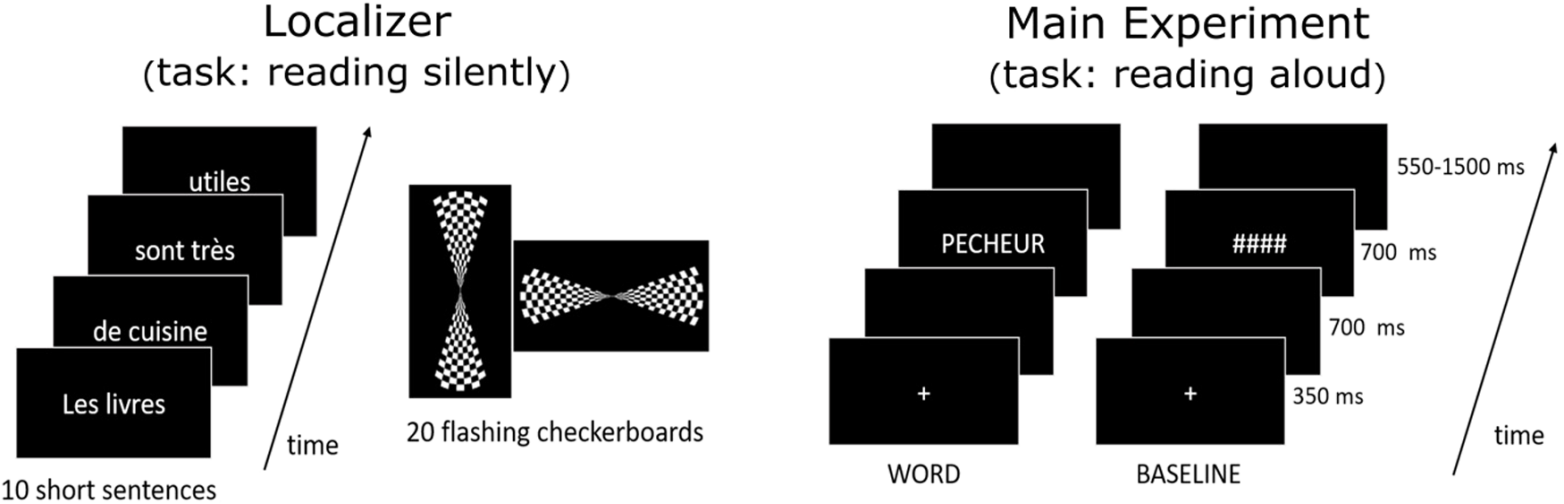
The Localizer task (left) consisted of 10 short sentences that had to be read silently and their activation as contrasted with viewing flashing checkerboards. The main reading task (right) consisted of reading isolated words out loud and their activation was contrasted with viewing meaningless symbols

We used two tasks, an fMRI Localizer task and the main reading task (see Fig. 4).

#### 2.3.1 Localizer task

The Localizer was an adaptation of a 5-min-long task introduced by Pinel et al. (2007). It captures the cerebral bases of auditory and visual perception, motor actions, reading, language comprehension, and mental calculation. Ten types of trials were mixed together and presented randomly: (1) passive viewing of flashing horizontal checkerboards (10 trials), (2) passive viewing of flashing vertical checkerboards (10 trials), (3) pressing the left button three times with the left thumb button according to visual instructions (5 trials), (4) pressing the right button according to visual instruction (5 trials), (5) pressing the left button three times according to auditory instruction (5 trials), (6) pressing the right button three times according to auditory instruction (5 trials), (7) silently reading short visual sentences (10 trials), (8) listening to short sentences (10 trials), (9) silently solving visual subtraction problems (10 trials), and (10) silently solving auditory subtraction problems (10 trials). Twenty rest periods (black screen) were inserted into the sequence and served as null events for a better hemodynamic deconvolution. Sentences were displayed as four successive screens (250 ms) separated by 100 ms interval, and each composed of a group of one to three words, resulting in 1.3 s of visual stimulation. Auditory stimuli were digitally recorded by a male speaker (resolution of 16 bits and sampling frequency of 22.05 kHz) and had a similar duration (1.2–1.7 s).

#### 2.3.2 Reading task

Participants were presented with a word or a series of symbols (condition “Baseline”). They were asked to read aloud each word as fast and correctly as possible. Baseline meaningless symbols (####) preserved the same number of characters as words. All stimuli were presented in white on a black background, with each letter subtending about 1.4° of visual angle. The words were displayed in Arial font at a size of 40 points. As can be seen in Figure 4 (right), we adopted a fast event-related design (see Kriegeskorte et al., 2008; Nili et al., 2014). The fixation cross was first presented in the center of the screen for ~350 ms, followed by a black screen of ~700 ms, then a stimulus (word or hash mark) of ~700 ms, and ended by a black screen jittered between ~550 and 1,550 ms. There were four runs, each consisted of 136 trials including 34 words repeated three times and 34 hash marks. Trials were presented pseudo-randomly in each run, and the order of the four runs was counterbalanced between subjects. The auditory and visual stimuli were managed and delivered using an in-house software developed in the NI LabVIEW environment (Bitter et al., 2017). The software was launched and real-time synchronized with the MR acquisition using an NI-PXI 6289 digital input/output hardware, which also allowed us to record the vocal and motor responses. The vocal responses in the MRI scanner were recorded using a FOMRI-II microphone (Optoacoustics Ltd., Or-Yehuda, Israel).

### 2.4 fMRI data acquisition

Data were collected on a 3-Tesla Siemens Prisma Scanner (Siemens, Erlangen, Germany) at the Marseille MRI centre (Centre IRM-INT@CERIMED) using a 64-channel head coil. Functional images (T2*-weighted gradient-echo planar sequence, 54 slices per volume, multi-band accelerator factor 3, repetition time = 1.23 s, spatial resolution = 2.5 x 2.5 x 2.5 mm, echo time = 30 ms, and flip angle = 65°) covering the whole brain were acquired during the reading tasks. Whole brain anatomical MRI data were acquired using high-resolution structural T1-weighted images (MPRAGE sequence, 256 slices, repetition time = 2.4 s, spatial resolution = 0.8 x 0.8 x 0.8 mm, echo time = 2.28 ms, and flip angle = 8°) in the sagittal plane. Prior to functional imaging, Fieldmap acquisition (Dual echo Gradient-echo acquisition, 54 slices per volume, repetition time = 7.1 s, spatial resolution = 2.5 mm^3^, echo time = 59 ms, and flip angle = 90°) was also collected in order to estimate and correct the B0 inhomogeneity. During the main experiment (reading task), a total of 1096 functional scans were acquired in four runs (4 x 274 scans). During the Localizer task, 256 functional scans were acquired in one run.

### 2.5 Data analysis

#### 2.5.1 fMRI data preprocessing

The fMRI data were pre-processed and analyzed using Statistical Parametric Mapping software (SPM12, http://www.fil.ion.ucl.ac.uk/spm/software/spm12/) on Matlab R2018b (Mathworks Inc., Natick, MA).

The anatomical scan was spatially normalized to the avg152 T1-weighted brain template defined by the Montreal Neurological Institute using the default parameters (nonlinear transformation). The fieldmap images were used during the realign and unwarp procedure for distortion and motion correction. Functional volumes were spatially realigned, normalized (using the combination of (i) deformation field, (ii) coregistered structural, and (iii) sliced functional images), resampled to an isometric voxel size of 2.5 mm and spatially smoothed by convolution of a Gaussian kernel of 5 mm full-width at half-maximum.

The Artifact Detection Tools (ART, http://www.nitrc.org/projects/artifact_detect/) were used to identify outlier volumes based on motion and on average signal intensity. Outliers were defined as any image where head placement deviated from the previous image in x, y, or z direction by more than 0.9 mm or whose average signal intensity differed from the series average by more than 5 standard deviations. Two participants were excluded from univariate analyses, because of excessive head movements during the acquisition. A total of 40 subjects (20 dyslexic and 20 control readers) were retained in this study.

### 2.6 Univariate analysis

#### 2.6.1 Localizer task

For each subject, a general linear model was generated for the complete design. It included 11 regressors of interest modelling the conditions of the Localizer (black screen, vertical checkerboard, horizontal checkerboard, left hand visual, left hand auditory, right hand visual, right hand auditory, calculation visual, calculation auditory, sentence visual, and sentence auditory). Six rigid-body realignment parameters (three translations and three rotations), one average signal intensity measure and the outliers detected by ART were included in the model as nuisance regressors to account for artifacts related to head motions and aberrant variations of signal intensity during the scanning. The amount of motion in all directions (Euclidian distance measure) was not different between groups (DYS: 0.48 +/- 0.56; CTR: 0.31 +/- 0.42, *p* > 0.296). The mean number nuisance parameters including outlier scans was also not different between the groups (DYS: 10.5 +/- 7.2, CTR: 9.0 +/- 3.4, *p* < 0.392).

Data were high-pass filtered with a cutoff of 128 s. Stimulus-specific BOLD effects were estimated by convolving the word-stimulus onsets with the canonical hemodynamic response function. To ensure maximal coverage of the anterior temporal lobes, an explicit masking threshold was set to 30% of the global anatomical brain reconstructed individually after the SPM segmentation (using tissues from normalized grey matter, white matter, and cerebrospinal fluid). We then focused only on the contrast between reading sentences versus viewing flashing checkerboards (Pinel & Dehaene, 2010; Pinel et al., 2007). This was done to define for each participant a functional region of interest that covered the whole reading network of a given participant.

##### 2.6.1.1 Main experiment

The analysis was based on the contrast between reading words versus hash marks to tap the reading network for both the dyslexic and typical readers. The GLM included, for each of the four runs, 5 regressors of interest, the noninterest regressors from ART (outliers scans from global signal and head movements), and one regressor for each run modelling the temporal mean of the signal. The amount of motion in all directions (Euclidian distance measure) was not different between groups (DYS: 0.45 +/- 0.33, CTR: 0.36 +/- 0.26, *p* < 0.34). The mean number of nuisance parameters including outlier scans was also not different between the groups (DYS: 10.18 +/- 6.79, CTR: 8.54 +/- 4.73, *p* > 0.38). The 5 regressors of interest were composed of the orthographically similar words from the “art” category (O+ART), the orthographically similar words from the “water” category (O+WATER), the orthographically dissimilar words from the “art” category (O-ART), the orthographically dissimilar words from the “water” category (O-WATER), and the hash mark condition (####). Regressors were convolved with the canonical hemodynamic response function (HRF), and the default SPM autoregressive model AR(1) was applied. Functional data were filtered with a 128 s high-pass filter. Statistical parametric maps for each experimental factor and each participant were calculated at the first level and then entered into a second-level one-sample *t*-test analysis of variance (random effects analysis or RFX using a threshold at the voxel level of 0.001 without correction for multiple comparisons). Whole-brain analysis results are displayed after controlling for the false discovery rate (FDR) at 0.05 for multiple comparisons at cluster level. Stereotaxic coordinates for voxels with maximal *t* values within activation clusters are reported in the MNI standard space.

### 2.7 Regions of interest (ROIs)

The regions of interest that belong to the reading network described by meta-analyses of neuroimaging studies (e.g., Jobard et al, 2003; Price, C. J. 2012) were obtained from the Localizer task (see Table S1, for a description of the significant clusters relative to the contrast “reading sentences versus viewing flashing checkerboards,” which allowed to identify the reading network for each participant). The ROIs were first created using the SPM Anatomy toolbox (Eickhoff et al., 2005) and WFU PickAtlas Standard Atlases (Maldjian et al., 2003). Figure 2A presents an overview of the ROIs on the cortical surface of a standard MNI brain. The following ROIs were selected: three subparts of inferior frontal gyrus (Brodmann areas BA44, BA45, and BA47, Amunts et al., 1999), one region of superior temporal gyrus and middle temporal gyrus (STG and MTG, Cavalli et al., 2016; Helenius et al., 1999; Price, 2012), and four subparts of the fusiform gyrus (FG1, FG2, FG3, and FG4, Caspers et al., 2013; Weiner & Zilles, 2016; see studies of Lerma-Usabiaga et al. 2018 and Vinckier et al. 2007 that confirmed the hierarchical organization of visual word processing along the ventral stream, with the posterior subpart involved in visual extraction and pure orthographic processing and the anterior subpart involved in integrating information with other regions of the language network). We used the left- and right-hemisphere analogues of these regions. All ROIs were converted into the native space of each subject using the inverse transformation matrix of magnetic field deformations (that was used to normalize the subject’s T1 image in the standard MNI space).

### 2.8 Representational similarity analysis (RSA)

Representational similarity analysis (RSA) was used to assess the neural representations of the words used in our study on orthographic and semantic dimensions (Kriegeskorte et al., 2006). That is, we used RSA to determine the regions that showed significant sensitivity to either orthographic or semantic similarity between word pairs, and this was done for the typical and dyslexic readers, separately.

### 2.9 BOLD response estimation

In order to take advantage of high spatial-frequency pattern information within each participants’ data in the RSA, we estimated condition-specific responses using a general linear model (GLM) based on functional native-space images unnormalized and unsmoothed. The GLM consisted of regressors of interest based on condition-specific image onsets convolved (one regressor for three repetitions of the same word) with a hemodynamic response function, and nuisance regressors based on GLMdenoise tools (Kay et al. 2013). For noise normalization, the estimated condition-specific GLM parameters were converted to *t*-values by contrasting each condition estimate against the implicitly modelled baseline (Charest et al., 2018). This resulted in 33 condition-specific *t*-value maps for each participant and each run (actually, 33 words * 4 runs).

### 2.10 Definition of neural RDMs

For each participant, images of the *t*-value maps masked with the 18 regions of interest were extracted using the CosMoMVPA toolbox (Oosterhof et al., 2016). For each participant and each ROI, we worked on a single trial basis of 132 samples (33 words * 4 runs). Voxels with no activation were removed from the sample-level data. Neural RDMs were computed for a set of voxels (within an ROI) using a cross-validated Euclidean distance (Leave-one-out cross-validation) between words from the covariance between samples (see Giordano et al., 2018). After averaging the cross-validated Euclidean distances across partitions (16 for 4*4 runs), the neural RDM resulted in a word-to-word matrix (33 x 33 words) for each subject at first-level analyses.

### 2.11 Definition of theoretical RDMs

Two theoretical RDMs were used in the RSA analysis (see Fig. 2B).

#### 2.11.1 Semmodel

This similarity matrix was based on calculation using a *t*-Distributed Stochastic Neighbor Embedding (*t*-SNE, see Hinton & Roweis, 2002; Van Der Maaten, 2014) that was trained on the French Wikipedia Corpus (Frwiki, 11 GB, 914,601,321 tokens). T-SNE is a nonlinear technique for dimensionality reduction that is extensively applied in image processing, genomic data, and speech processing.

#### 2.11.2 Orthmodel

This model was based on the Levenshtein distance between two words, which refers to the minimum number of editing operations (including replacing one character with another, inserting a character, and deleting a character) required to convert one word to another. We weighted shared initial and final positions more than shared middle positions (Grainger et al., 2016).

### 2.12 Definition of the confounding matrices

The confounding matrix was used to improve the signal-to-noise ratio during correlation calculation (Snoek et al., 2019). The confounding matrix was based on the average of the lexical frequency and word length matrices, which were two confounding factors known to interact with reading processes.

### 2.13 Comparing neural representations with theoretical models of semantic and orthographic similarity

RSA involves computing a second-order correlation (typically Pearson’s correlation) between theoretical RDMs and neural RDMs (Kriegeskorte et al., 2006, 2008; Mur et al., 2009). In our study, we used Spearman’s correlations, which unlike Pearson’s correlations, do not assume a linear link between the two RDMs. Moreover, we computed Spearman’s partial correlations using the two confounding matrices (lexical frequency and word length RDMs) to improve the signal-to-noise ratio.

To evaluate the significance level of the correlation between a given theoretical RDM and neural RDM, we used Wilcoxon signed rank tests. For a given ROI, two-sided signed rank tests were used both within or between subjects. Within a group of subjects, the correlation vector for each ROI was compared to a distribution whose median is zero. With the resultant *p*-values, we constructed a vector P of multiple-test false positive levels to correct the *p*-values for multiple comparisons on the theoretical RDM dimension. We used the FDR function (FDR for False Discovery Rate) from the CONN toolbox (www.nitrc.org/projects/conn) to transform the vector P to a vector Q of estimated false discovery rates. In the RSA results section, we will use the term Q-values instead of “corrected P-values”.

## 3 Results

### 3.1 Reading aloud task (in the scanner)

We analyzed only reading aloud latencies (RTs) for both groups given that the mean accuracy was at ceiling (99.9% for the typical readers and 99.4% for adults with dyslexia). Outliers with 2.5 standard deviations above and below the mean RT were deleted for each participant with no significant difference between groups (*t*(37) = 0.09; *p* = 0.93). The results of an ANOVA on mean RTs showed a significant main effect of group (F(1, 37) = 5.37; *p* = 0.02). The mean RT for the dyslexic readers was 621 ms and that of the typical readers was 552 ms. The results also showed a main effect of repetition (F(11, 407) = 2.86; *p* < 0.001), but the interaction between the effects of group and repetition was not significant (F(11, 407) = 0.81; *p* = 0.63). A fine-grained analysis of the repetition effect in the same data both at behavioral and neural levels is reported in Tan et al. (2022).

### 3.2 Univariate analysis on whole brain

**Fig. 5.**
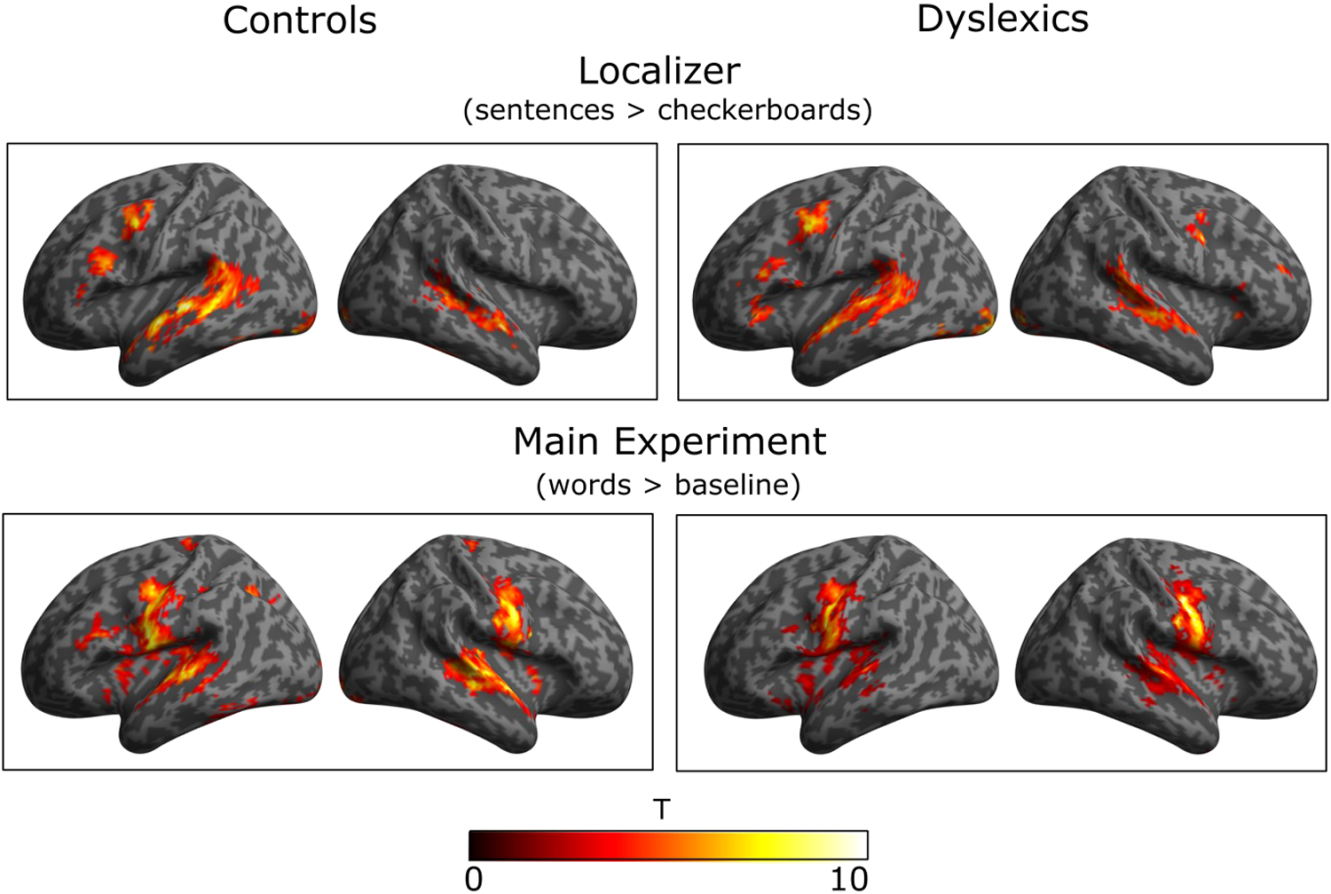
Statistical T-maps for each group of participants (20 typical readers on the left side and 20 dyslexic readers on the right side of the figure) were projected on an MNI cortical surface (left and right view within each framework). Activations correspond to significant differences of “reading sentence versus viewing checkerboards” in the case of the Localizer and “reading words aloud versus baseline” in the case of the main experiment [cluster threshold of *p* < 0.05, FDR corrected; MNI=Montreal Neurological Institute]. No statistical difference between the two groups of participants was revealed in either task (Localizer and main experiment).

Random effect analyses (RFX) were performed with SPM12 for the whole brain analyses for each group of participants (20 typical and 20 dyslexic readers). Figure 5 presents all activated regions significant at the cluster level with an FDR (False Discovery Rate) correction for multiple comparisons (see Tables S1 and S2 for the Localizer task and Tables S3 and S4 for the main experiment).

In the Localizer task, the “sentence minus checkerboard” contrast revealed a very similar activation profile for the two groups of participants: middle temporal lobe bilaterally, inferior occipital lobe and cerebellar regions, as well as frontal regions (precentral, inferior, and post-middle regions) on the left hemisphere (see upper panel of Fig. 5, *p* < 0.05 at cluster level with FDR correction and *p* < 0.001 without correction at voxel level). It is worth noting that the frontal activations in the right hemisphere were present only in the dyslexic group. However, when the two groups were compared statistically (using a two-sample *t*-test), no significant difference was found in any of the regions. In conclusion, the results did not show a significant difference between dyslexic and typical readers in silent sentence reading.

In the main experiment, the “reading words aloud minus baseline” contrast showed a very similar activation profile for the two groups of participants. As in the Localizer task, a left fronto-temporo-occipital network was activated, with a more pronounced activation in the premotor region bilaterally (see the lower panel of Fig. 5, *p* < 0.05 at the cluster level with FDR correction and *p* < 0.001 without correction at the voxel level). Visual inspection seems to suggest that the typical readers showed greater activation in left IFG and left middle temporal gyrus than dyslexic readers, but there was no significant difference between the two groups (for details see Tables S3 and S4). In sum, the results showed that university students with dyslexia activated the classic reading network when silently reading sentences or reading single words aloud. In the univariate analysis, this network was not different to that of typical readers.

### 3.3 Representational similarity analysis (RSA)

First, we will report the RSA results for the semantic dimension, including the ROIs, which showed a significant second-order correlation between the theoretical RDM and the neural RDM. We present the results for all participants, for each group separately, and the difference between the two groups. Second, we will show the same results for the orthographic dimension.

### 3.4 Semantic dimension

**Fig. 6.**
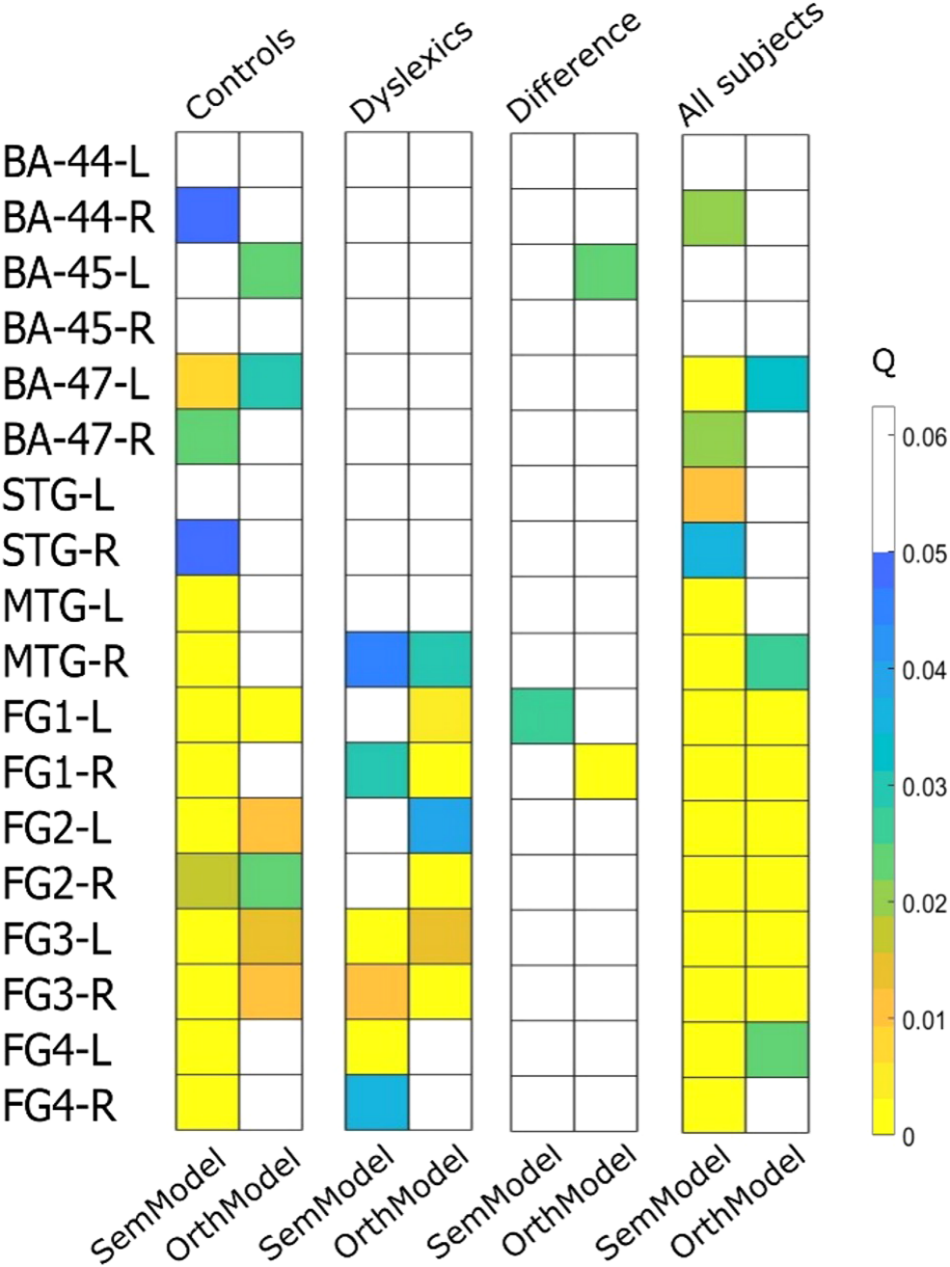
Results of Wilcoxon signed rank tests (Q-Values) to assess the correlations between the neural representational dissimilarity matrix (RDM) and two theoretical RDMs (SemModel: semantic; OrthModel: orthographic) for typical readers (“Controls”), dyslexic readers (“Dyslexics”), between the two groups (“Controls/Dyslexics”), and for all the subjects combined (“All subjects”). [BA = Brodmann area; STG = superior temporal gyrus; MTG= middle temporal gyrus; FG = fusiform gyrus].

**Fig. 7.**
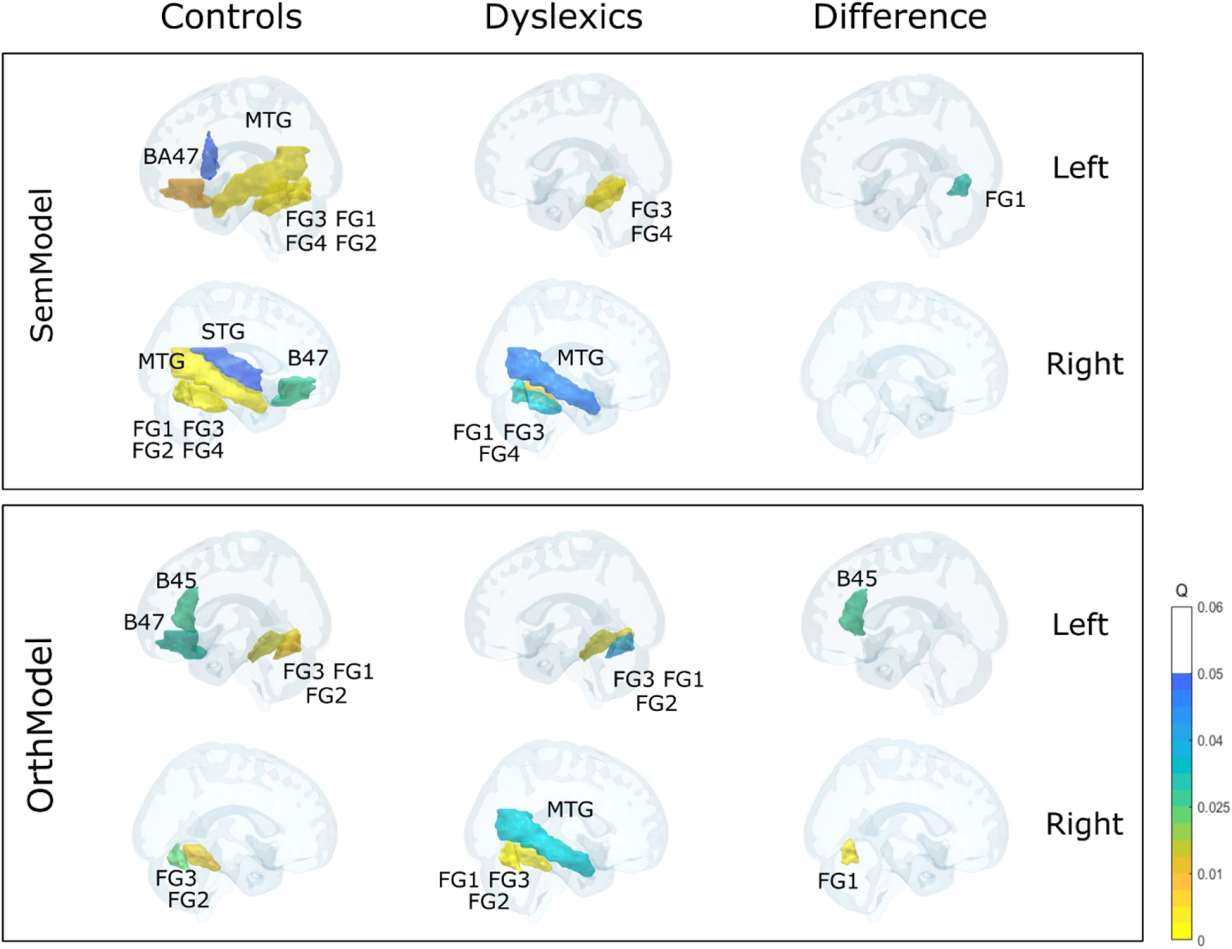
Results of the RSA analysis. Regions that showed significant correlations (Q-Values < .05) between the neural and the semantic (SemModel) representational dissimilarity matrix (RDM) are presented in the upper panel. Regions that showed significant correlations between the neural and the orthographic (OrthModel) RDM are presented in the lower panel. [BA = Brodmann area; STG = superior temporal gyrus; MTG = middle temporal gyrus; FG = fusiform gyrus].

The results of the RSA analysis are presented in Figure 6. As can be seen in this figure, semantic information was distributed in a large network for typical readers, which included the orbital part of inferior frontal gyrus in both hemispheres [q(BA-47-L) < .01, q(BA-47-R) <.03], the superior temporal gyrus in the right hemisphere [q(STG-R) < .05], the middle temporal gyrus both hemispheres [q(MTG-L) < .001, [q(MTG-R) < .001], all fusiform ROIs in both hemispheres [q(FG1-L) < .0001, q(FG1-R) < .0003, q(FG2-L) < .0002, q(FG2-R) < .02, q(FG3-L) < .0001, q(FG3-R) < .0001, q(FG4-L) < .0009, q(FG4-R) < .0007], and the opercular part of inferior frontal gyrus restricted to the right hemisphere [q(BA-44-R) < = .05]. This pattern of activation can also be seen in Figure 7 (the two figures on the left side of the upper panel).

Relative to typical readers, dyslexic readers displayed a more restricted network of brain regions that was sensitive to semantic information, including the right middle temporal gyrus, [q(MTG-R) < .05)], and the fusiform gyrus (restricted to FG1 in the right hemisphere and FG3 plus FG4 in both hemispheres, [q(FG1-R) < .03, q(FG3-L) < .0001, q(FG3-R) < .001, q(FG4-L) < .003, q(FG4-R) < .04] (see Fig. 6, results of SemModel for “Dyslexics”, and Fig. 7, the two figures in the middle of the upper panel). A direct comparison between the two groups using a two-sample *t*-test showed that the left FG1 fusiform was the only region that exhibited a statistically significant difference between dyslexic and typical readers [q(FG1-L) < .03] (see Fig. 6, results of SemModel for “Controls/Dyslexics,” and Fig. 7, the two figures on the right side of the upper panel). Specifically, the left FG1 was more sensitive to semantic information/similarity in typical readers than in dyslexic readers.

### 3.5 Orthographic dimension

The same analyses were conducted for the orthographic dimension. Across all participants, orthographic information was represented in the three fusiform ROIs bilaterally: bilateral FG1, FG2, and FG3 [q(FG1-L) < .0001, q(FG1-R) < .0007, q(FG2-L) < .0009, q(FG2-R) < .0001, q(FG3-L) < .0002, q(FG3-R) < .0001)] (see Fig. 6, results of OrthModel for “All subjects”). For dyslexics, it also included bilateral FG1, FG2, and FG3 [q(FG1-L) < .005, q(FG1-R) < .0001 q(FG2-L) < .04, q(FG2-R) < .0002, q(FG3-L) < .02, q(FG3-R) < .0003] (see Figs. 6 and 7). The control group showed sensitivity to orthographic information in inferior frontal gyrus in the left hemisphere [q(BA-45-L) < .03, q(BA-47-L) < .03], fusiform gyrus FG1 in the left hemisphere [q(FG1-L) < .002], and bilateral FG2 and FG3 [q(FG2-L) < .02, q(FG2-R) < .03, q(FG3-L) < .02, q(FG3-R) < .02] (see Figs. 6 and 7). Significant differences between the RSA results of the two groups were obtained in the left Broca_45 area [q(Broca-45-L) < .03] and right FG1 fusiform region [q(FG1-R) < .003) (see Figs. 6 and 7). These differences reflected the fact that the left inferior frontal gyrus (BA-45) was more sensitive to orthographic information/similarity in typical readers than in dyslexic readers.

### 3.6 Direct comparison between orthographic and semantic similarity of left and right FG1 and BA45

To assess more directly representational differences between the two groups on both semantic and orthographic dimensions in the two key regions that showed differences, we conducted a two-way ANCOVA on the second order of correlation (correlation between neural RDM and theoretical RDM) with Group (Dyslexics vs. Controls) and Model (SemModel vs. OrthoModel) as factors and Reading fluency as a covariate in order to account for inter-individual differences in reading fluency within each group. This analysis was done for the three main ROIs that showed significant group differences.

**Fig. 8.**
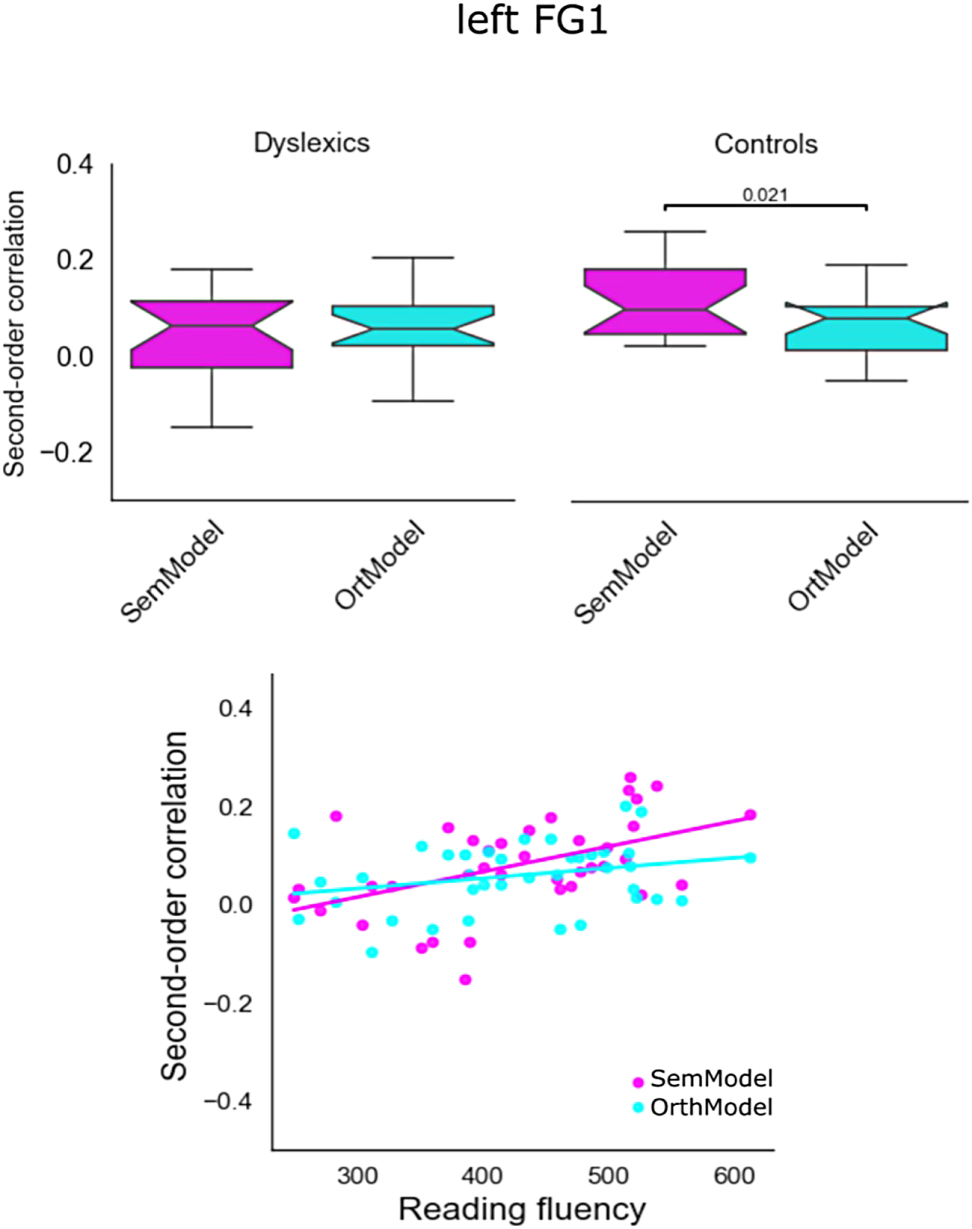
Second-order correlations in left FG1 for the model (semantic and orthographic) dimension for both groups (upper part) and individual second-order correlations for the model dimension as a function of reading fluency.

For the left FG1, the results of ANCOVA revealed a main effect of Group (F(1,68) = 6.65, *p* < 0.05), a main effect of the Reading fluency (F(1,68) = 7.0, *p* < 0.05), and a significant Group by Model interaction (F(1,68) = 4.21, *p* < 0.05). The results are presented in Figure 8. The boxes in the upper part of the figure show the quartiles of the dataset (and the box notch the median) per model and per group. The lower part of the figure shows the second order of correlations for semantic and orthographic similarity for all participants as a function of their reading fluency.

In left BA45, the results of ANCOVA revealed a main effect of Group (F(1,28) = 4.95, *p* < 0.05) but the Group by Model interaction failed to reach significance (F(1,28) = 3.11, *p* < 0.1). Results are presented in Figure S1.

**Fig. 9.**
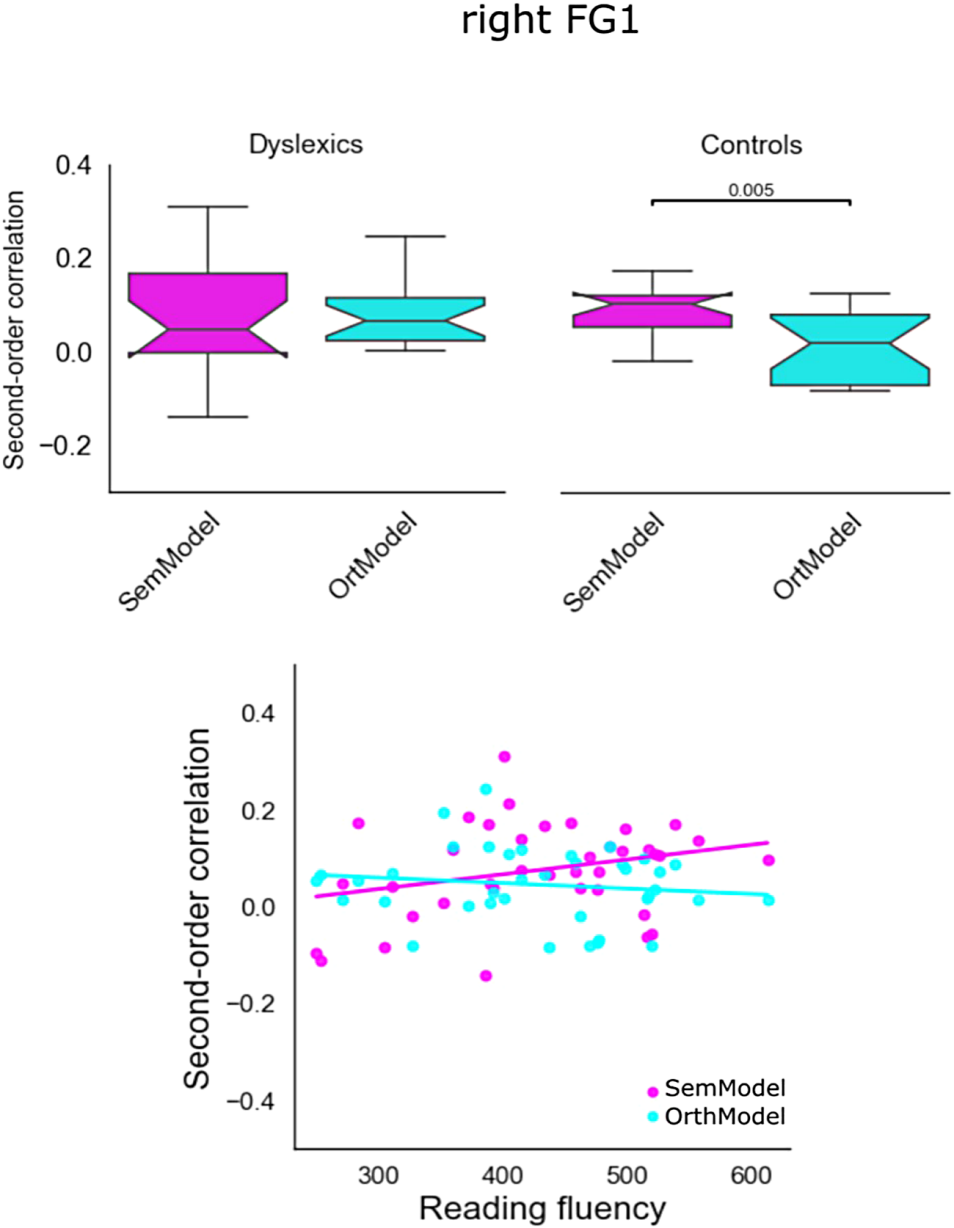
Second-order correlations in right FG1 for the model (semantic and orthographic) dimension for both groups (upper part) and individual second-order correlations for the model dimension as a function of reading fluency.

For the right FG1, the results of ANCOVA revealed a main effect of Reading fluency (F(1,68) = 5.89, *p* < 0.05) and a significant Group by Model interaction (F(1,68) = 4.21, *p* < 0.05). The results are presented in Figure 9. The boxes in the upper part of the figure show the quartiles of the dataset (and the box notch the median) per model and per group. The lower part of the figure shows the second order of correlations for semantic and orthographic similarity for all participants as a function of their reading fluency.

### 3.7 Posterior-to-anterior gradient of orthographic and semantic processing

To test the hypothesis according to which the ventral stream (FG1, FG2, FG3, and FG4) is organized according to a posterior-to-anterior gradient that represents increasingly higher level linguistic information (i.e., semantics), an additional ANOVA was conducted on the mean second-order correlations with Group (Dyslexics vs. Controls), Model (SemModel vs. OrthoModel), and Gradient (posterior left FG1 and FG2 vs. anterior left FG3 and FG4) as factors. The results of the ANOVA revealed a main effect of Group (F(1,296) = 4.28, *p* < 0.05), a main effect of Model (F(1,296) = 19.6, *p* < 0.001), a significant Gradient by Model interaction (F(1,296) = 4.8, *p* < 0.05) and the Gradient by Group and Gradient by Group by Model interaction were only close to be significant (F(1,296) = 3.66, *p* = 0.056 and F(1,296) = 3.79, *p* = 0.052, respectively). Post hoc comparisons (Tukey correction) showed no significant difference between semantics and orthography in posterior left FG1 and FG2 (*p* = 0.40), but clear difference was present in left anterior FG3 and FG4 (*p* < 0.001), which was more sensitive to semantic information/similarity than to orthographic information/similarity.

## 4 Discussion

The goal of the present study was to investigate whether we could find direct evidence for compensatory re-organization of the reading network in high-functioning adults with dyslexia. Indeed, it has been suggested that university students with dyslexia use higher level linguistic information, in particular semantics, to compensate for lower-level orthographic and phonological processing deficits (Cavalli et al., 2016; Snowling, 2000; Stanovich, 1980). However, previous brain imaging results in favor of this hypothesis were rather mixed. For example, some studies reported greater activation of the left IFG, a region involved in speech production and semantic processing, in dyslexics than in controls (Brunswick et al., 1999; Grünling et al., 2004; Shaywitz & Shaywitz, 2005; Shaywitz et al., 1998), while other studies showed no differences between the two groups (Eden et al., 2004; Rumsey et al., 1994) or even lower levels of activation in dyslexics than in controls (Brambati et al., 2006; Georgiewa et al., 1999; Paulesu et al., 1996; Rumsey et al., 1997). In addition, activation-based analyses are somewhat limited because the overall level of activation of an area does not necessarily tell us much about what kind of information is being processed and greater levels of activation do not necessarily mean “more” or “better” linguistic processing. Finally, it is difficult to dissociate the linguistic compensatory hypothesis from an “increased cognitive effort” hypothesis, which suggests that increased effort of adults with dyslexia would be responsible for univariate whole-brain activation differences.

To obtain direct evidence for representational differences in the reading network of university students with dyslexia, we applied an MVPA RSA method (Kriegeskorte et al., 2008) to find out which regions of the reading network are in charge of processing orthographic and semantic information and whether there were any differences between typical and dyslexic readers. In accordance with the compensatory hypothesis, we predicted that adults with dyslexia might show greater second-order correlations between the semantic (dis)similarity of words and the (dis)similarity of neural responses to these words in regions that care about semantics and weaker second-order correlations between the orthographic (dis)similarity of words and the (dis)similarity of neural responses to these words in regions that care about orthographic processing.

The results can be summarized as follows: First, the reading level assessment and the results of the reading aloud task in the scanner clearly showed that when compared to the typical readers, university students with dyslexia performed more poorly on all reading and reading-related tasks with weaker ARHQ-R scores, weaker reading fluency, and phonological processing efficiency scores, as well as slower RTs in reading aloud isolated words. The range of effects sizes varied from 0.9 to 3.3 (Cohen’s *d*) indicating that the size of the deficit is medium to large. It is important to note that all the participants with dyslexia in the present study have (1) received a formal diagnosis of dyslexia during primary school, (2) reported having experienced major difficulties in reading from childhood to adulthood, and (3) received remedial teaching by a specialized speech therapist from childhood to adolescence. Importantly, the two groups did not significantly differ on verbal IQ as assessed by the standardized EVIP vocabulary task (all the participants scored above the fifth percentile) thereby confirming that none of the participants presented a deficit in oral language skills (see Cavalli et al., 2016). These behavioral findings clearly confirm that while our group of adults with dyslexia successfully managed to study at the university level, they still presented reading and phonological processing impairments, which constitute a hallmark of developmental dyslexia in adults (Brèthes et al., 2022; Cavalli, Colé et al., 2017; Lefly & Pennington, 2000; Martin et al., 2010).

Second, the univariate analysis of the Localizer and the reading task activated the classic reading network (Rueckl et al., 2015) in both reader groups and there were no statistically significant differences between them (we will come back to this finding below). Third, the RSA analyses showed that the entire ventral stream, that is, all fusiform gyrus subparts (FG1, FG2, FG3, and FG4) bilaterally, was sensitive to semantic information in typical readers, whereas less sensitivity to semantic information was obtained for dyslexic readers in the posterior subpart of the ventral stream (left FG1). Specifically, reading fluency (as a continuous variable) predicted the sensitivity of FG1 to semantic information/similarity (better readers show greater sensitivity to semantic information, see Fig. 8). Fourth, in typical readers, orthographic information was not only processed in the left fusiform gyrus (FG1, FG2, and FG3) but also in left IFG. Adults with dyslexia did not show sensitivity to orthographic information in left IFG. However, they showed increased sensitivity to orthographic information in the right FG1 (see Fig. 9). Interestingly, reading fluency (as a continuous variable) was negatively related to the sensitivity to orthographic information/similarity in the right FG1 suggesting that poorer readers rely to a greater extent to orthographic processing in the right hemisphere homologue of FG1.

Together, the results show atypical orthographic processing in left IFG and right FG1 and reduced semantic information in left FG1. While there is evidence for some level of re-organization in adults with dyslexia, the present results do not support the hypothesis according to which adults with dyslexia use higher level semantic information more efficiently (i.e., greater correlations of neural RDM in ROIs associated with semantic processing). The results will further be discussed below.

### 4.1 Univariate analyses of the fMRI data

In terms of univariate differences between adult dyslexic and typical readers, some previous studies have shown reduced activation in left inferior frontal gyrus (Brambati et al., 2006; Devoto et al., 2022; Georgiewa et al., 1999; Paulesu et al., 1996; Rumsey et al., 1997) and the left middle temporal gyrus in dyslexic readers (Cao et al., 2006; Grünling et al., 2004; Kronbichler et al., 2006; Meyler et al., 2007; Paulesu et al., 2001), which could be taken to suggest that these regions are less well tuned to process written words. Other studies found overactivation of left IFG (Brunswick et al., 1999; Devoto et al., 2022; Grünling et al., 2004; Shaywitz & Shaywitz, 2005; Shaywitz et al., 1998), which could be interpreted as increased effort. In our study, we found no differences between the two groups of readers in the two reading tasks. This result was expected because we deliberately chose “easy” reading tasks such that performance differences between the groups could be excluded (see Ingvar et al., 2002). Moreover, we used a paradigm in which words were repeated 12 times across four runs intermixed with hash marks. Therefore, our reading task included massive repetition of the same words and it has been previously shown that three repetitions of the same words are sufficient for dyslexic readers to show “normal” activation of the left-hemisphere reading network compared to typical readers (Pugh et al., 2008). In support of this interpretation is an analysis of the same dataset by Tan et al. (2022) who showed clear evidence for significant repetition effects (i.e., neural adaptation) in left fusiform gyrus for dyslexic readers. However, when compared to typical readers, there was no evidence for greater levels of variability to repeated presentations of the same stimulus neither in the behavioral nor the neural responses. This is in line with the finding of Beach et al. (2022) who found repetition effects in adults with dyslexia that were not different from those of typical readers.

It should be noted, however, that the dyslexic readers in our study were university students who benefited from many years of reeducation, which might have compensated for potential deficits in single word reading (Cavalli, Colé, et al., 2017). In support of this idea, many remediation studies on individuals with dyslexia have shown that reading improvements subsequent to interventions were accompanied by a substantial increase of the activation level in the left occipitotemporal cortex during reading (e.g., Brem et al., 2010; Heim et al., 2015). On the positive side, the seemingly disappointing absence of a global activation differences between groups implies that subtle differences in the re-organization of the reading network (i.e., our results from the RSA analysis) could not be imputed to differences in the absolute levels of activation, which could have been due to less processing efficiency or increased effort.

### 4.2 Multivariate analysis (RSA) of the fMRI data

The RSA results for the semantic dimension showed that semantic information in both typical and dyslexic readers was represented along the entire ventral stream of word processing including bilateral inferior frontal gyrus and fusiform gyrus (Cohen & Dehaene, 2009; Sandak et al., 2004) and also along the dorsal stream including bilateral superior and middle temporal gyrus (Jobard et al., 2003; Price, 2012). This result indicates that the processing of semantic information is widely distributed (Huth et al., 2016) and involves the cooperation of all the fusiform brain regions related to visual word reading (Cohen & Dehaene, 2009; Sandak et al., 2004) and the temporal brain regions related to auditory association and multisensory integration (Jobard et al., 2003; Price, 2012). Previous studies have confirmed the hierarchical organization of visual word processing along the ventral stream, with the posterior subpart involved in visual extraction and pure orthographic processing and the anterior subpart involved in integrating information with other regions of the language network (Lerma-Usabiaga et al., 2018; Vinckier et al., 2007). This posterior-to-anterior word similarity gradient of the visual word form area (VWFA) is predicted by Dehaene et al.’s hierarchical model of word recognition (Dehaene et al., 2005) and recently confirmed in a high-resolution fMRI study (Zhan et al., 2023). Our RSA results are consistent with this model as all the fusiform gyrus subparts (FG1, FG2, FG3, and FG4) were sensitive to semantic information, whereas only the more posterior subparts (FG1, FG2, and FG3) were also sensitive to orthographic information, suggesting that more integrated semantic word processing takes place in the anterior fusiform gyrus.

These results are consistent with a hierarchical organization of visual processing along the ventral stream with posterior-to-anterior gradient reflecting more integrated and semantic word processing in anterior parts (left FG3 and FG4) with typical readers tend to show greater level of both orthographic and semantic similarity than dyslexics readers. However, findings from a recent MEG study conducted in adults with dyslexia support the hypothesis that the left FG exhibits an altered posterior-to-anterior gradient, which suggests a spatiotemporal re-organization of the ventral stream (Cavalli, Colé, et al., 2017). Arguably, it also suggests that the visual word form system is doing much more than processing orthographic features but rather integrating the bottom-up visual and orthographic information with higher level associations, such as meanings (see Price & Devlin, 2003, 2011). In fact, different systems can share information between them as revealed in other RSA studies (e.g., Carota, Colé, et al., 2017; Pegado et al., 2018). Contrary to the prediction made by the compensatory re-organization hypothesis, the RSA revealed that the left FG1 is less sensitive to semantic information in dyslexic readers (i.e., weaker second-order correlation, see Fig. 8). Although the finding is consistent with many previous studies, which reported less activation in the fusiform area in adults with dyslexia (Brambati et al., 2006; Brunswick et al., 1999; Maisog et al., 2008; McCrory et al. 2005; Paulesu et al., 2001), it also suggests that left FG1 of adults with dyslexia is less well tuned for integrating semantic information within the bottom-up orthographic stream.

The RSA results for the orthographic dimension showed that orthographic information in both typical and dyslexic readers was mainly represented along the ventral stream of word processing including bilateral fusiform gyrus (FG1, FG2, and FG3), and also in left inferior frontal gyrus (left BA45) in typical readers. Although most previous studies have shown that the left FG is particularly sensitive to orthography (Cohen et al., 2000; Devlin et al., 2006; McCandliss et al., 2003), there is also evidence for the involvement of the left IFG in orthographic processing. First, using MEG, Cornelissen et al. (2009) showed that the left IFG was activated during the first 200 ms during a silent word reading task. Second, using fMRI, Montant et al. (2011) showed that lexical orthography, which is needed to discriminate a pseudohomophone from its base word (BRANE-BRAIN), is represented in left IFG (see also Braun et al., 2015, for a similar finding). Interestingly, our RSA analyses showed that the left IFG (BA45) was more sensitive to the orthographic similarity between words in typical readers than in dyslexic readers. For dyslexic readers, in contrast, the RSA revealed that orthographic similarity was represented in the right-hemisphere analogue of FG1 (i.e., stronger second-order correlations). A previous study, in which participants had to learn novel words in an artificial script, suggested that efficient reading strategies (i.e., focusing on the grapheme-phoneme structure of novel words) produced left-lateralized responses of the fusiform gyrus (Yoncheva et al., 2015), whereas inappropriate reading strategies (i.e., memorizing the novel words as a whole) showed bilateral activation of the fusiform gyrus. Thus, the right-hemisphere activation of the FG in response to orthographically similar words might reflect the remnants of the reading difficulties and possibly inefficient reading strategies encountered during childhood. Note also that Ingvar et al. (2002) found more activation in the right occipitotemporal cortex in dyslexics than controls. Contrary to the prediction made by the compensatory re-organization hypothesis, the RSA revealed that the right FG1 was more sensitive to orthographic information in dyslexic readers. This result is consistent with white matter connectivity studies showing hyperactivation in the right hemisphere analogue of the left occipitotemporal visual word form area and negative correlation between the right FG and reading skills in the dyslexia group (Liu et al., 2021; Pugh et al., 2000; Zhao et al., 2017).

### 4.3 Perspectives on compensation in adults with dyslexia and conclusion

In summary, while we found evidence for compensatory re-organization in adult dyslexia, the present results do not support the hypothesis according to which adults with dyslexia rely more heavily on semantic information. Instead, they revealed atypical hemispheric organization and more specifically a lack of sharing semantic and orthographic information between key regions of the reading network that are more heavily dedicated to speech production (IFG) and orthographic processing (FG). Indeed, the present findings suggest that high-functioning adults with dyslexia seem to be less able to integrate semantic information during orthographic processes (left FG1). They also seem to be impaired in processing orthographic similarity in left IFG and the more severely impaired dyslexics (weaker reading fluency scores) seem to rely on right-homologues of the fusiform gyrus to process orthographic information. Interestingly, some researchers interpreted the positive correlation between neural underpinnings and reading performance as a compensatory mechanism for children with dyslexia (Hoeft et al., 2011), but previous fMRI studies in children with dyslexia as well as the current fMRI study in high-functioning adults with dyslexia observed negative association between brain response patterns or neural activity and reading performance. These patterns of results might indicate a maladaptive compensatory mechanism toward orthographic processing or a compensatory orthography-to-semantics reading route (Seidenberg & McClelland, 1989), as suggested by Liu et al. (2021) who reported negative correlation between right FG white matter connectivity and pseudoword reading accuracy in children with dyslexia. Nevertheless, caution should be exercised when comparing results from studies conducted on children or adults with dyslexia. Moreover, there is currently no agreed-upon technical definition of compensation that would describe its behavioral, cognitive, and neural characteristics. Indeed, the use of the term “compensatory mechanism” has thus far been unclear and ambiguous mainly due to vague definition used in the literature (Fleming et al., 2018; Hancock et al., 2017). Following Livingston and Happé (2017), at the neural level, compensation is associated with a change difference in neural activity or pathways outside the networks typically involved in the cognitive operation of interest (here reading) that serves to facilitate behavioral performance of individuals with dyslexia (Hoeft et al., 2011).

One limitation of the present study is that it looked only at the spatial response patterns in relation to orthographic and semantic information. Such analyses ignore potential differences in the relative *timing* of the mechanisms. This has been suggested in a study by Cavalli, Colé, et al. (2017) who used MEG in a primed lexical decision task and found that morpho-semantic priming occurred earlier in the left inferior frontal gyrus (left BA45) than orthographic priming in adults with dyslexia, while the opposite was true for orthographic priming. Cavalli et al.’s findings suggested that the stronger reliance on morpho-semantic processes could be seen as one of the neural signatures for compensation. The methods presented in the present study can be easily applied to the spatiotemporal domain (see Lu et al., 2015) and would certainly provide interesting additional insights into the temporal and spatial tuning of the reading network of people how face dyslexia.

## Supplementary Material

Supplementary Material

## Data Availability

The analysis code and data related to this article are openly available at OpenNeuro (https://openneuro.org/datasets/ds004786/versions/1.0.1; OpenNeuro Accession Number: ds004786).

## Footnotes

1
Warmington et al. (2013) estimated that that approximately 3.2% of the dyslexics in the UK manage to undertake university studies despite having dyslexia.
