# Supplementary material for: Atypical hemispheric re-organization of the reading network in high-functioning adults with dyslexia: Evidence from representational similarity analysis
Source: Imaging Neurosci (Camb). 2024 Jan 22;2:imag-2-00070. doi: 10.1162/imag_a_00070 (PMC12224412; doi:10.1162/imag_a_00070)
Supplement: Supplementary Material [file imag_a_00070-supp.pdf]

Supplementary materials

Table S1. *Localizer – Contrast 'Reading sentences versus Viewing flashing checkerboards* for the control group. Statistical results of whole brain univariate analysis (T-value and Z-value were computed at voxel-level). All peaks were significant at an uncorrected voxel-wise threshold of p < .001 and a (Family-Wise Error) corrected cluster-wise threshold with of p < .05. Regions were automatically labelled using the Neuromorphometrics atlas (http://neuromorphometrics.com/). Montreal Neurological Institute coordinates (MNI coords) were listed in millimeters in the left-right (X), posterior-anterior (Y), and inferior-superior (Z) dimensions. [L = left; R = right]

			MNI coords			
T-value	Z-value	Cluster size	X	Y	Z	Region label of main cluster
10.41	5.95	1485	-55	-29	-2	L Superior Temporal Gyrus
10.21	5.89		-58	-9	-7	
9.18	5.61		-55	-44	3	L MiddleTemporal Gyrus
9.14	5.60	628	53	-11	-12	R Superior Temporal Gyrus
7.88	5.19		48	-39	6	R MiddleTemporal Gyrus
6.66	4.73		58	-26	1	R Superior Temporal Gyrus
8.73	5.47	351	-48	2	43	L Precentral Gyrus
7.00	4.87		-40	-4	51	
6.91	4.83		-40	-4	38	
7.17	4.93	303	-43	-31	-20	L Fusiform Gyrus
5.78	4.34		-43	-49	-20	
5.61	4.25		-50	-61	-25	L Cerebellum Ext
6.84	4.80	203	-45	14	23	L Inferior Frontal Gyrus (p. Opercularis)
5.94	4.42		-35	19	21	L Middle Frontal Gyrus
8.08	5.26	161	-10	12	53	L Supplementary Motor Cortex
7.73	5.14	132	-35	-86	-17	L Inferior Occipital Gyrus
5.92	4.40		-30	-91	-12	
5.40	4.15		-23	-96	-10	L Fusiform Gyrus
6.10	4.48	101	18	-79	-37	R Cerebellum Ext
5.11	4.01		10	-79	-45	
8.11	5.27	71	28	-99	-10	R Occipital Pole
5.00	3.95	46	-50	29	8	L Inferior Frontal Gyrus (p. Triangularis)
4.77	3.82		45	-64	-27	R Cerebellum Ext
4.61	3.73		33	-61	-30	

Table S2. *Localizer – Contrast 'Reading sentences versus Viewing flashing checkerboards* for the dyslexic group. Statistical results of whole brain univariate analysis (T-value and Z-value were computed at voxel-level). All peaks were significant at an uncorrected voxel-wise threshold of $p < .001$ and a (Family-Wise Error) corrected cluster-wise threshold with of $p < .05$. Regions were automatically labelled using the Neuromorphometrics atlas (http://neuromorphometrics.com/). Montreal Neurological Institute coordinates (MNI coords) were listed in millimeters in the left-right (X), posterior-anterior (Y), and inferior-superior (Z) dimensions. [L = left; R = right]

			MNI coords			
T-value	Z-value	Cluster size	X	Y	Z	Region label of main cluster
8.79	5.49	1621	-58	-6	-10	L Superior Temporal Gyrus
8.23	5.31		-65	-29	1	
7.65	5.11		-53	-44	6	L Middle Temporal Gyrus
7.35	5.00	904	58	-6	-10	R Superior Temporal Gyrus
8.43	5.38		58	-19	-5	
7.96	5.22		55	-41	8	R Middle Temporal Gyrus
8.59	5.43	593	-48	4	51	L Precentral Gyrus
8.15	5.28		-38	-1	58	
8.85	5.51		-48	-4	41	
5.25	4.07	379	8	19	43	R Supplementary Motor Cortex
6.19	4.52		-8	12	51	L Supplementary Motor Cortex
9.50	5.70		-3	7	63	
7.06	4.89	305	-45	-71	-15	L Inferior Occipital Gyrus
10.36	5.93		-33	-91	-17	
9.83	5.79		-25	-96	-12	L Occipital Fusiform Gyrus
9.07	5.58	204	48	4	38	R Precentral Gyrus
6.17	4.52		40	4	58	R Middle Frontal Gyrus
5.85	4.37	204	45	4	51	R Precentral Gyrus
7.99	5.23	199	-5	-74	-22	L Cerebellum Ext
7.13	4.92		5	-74	-27	R Cerebellum Ext
6.25	4.55		-3	-79	-37	L Cerebellum Ext
9.85	5.80	191	30	-61	-27	R Cerebellum Ext
4.60	3.72	110	33	-89	-15	R Occipital Fusiform Gyrus
8.69	5.46		28	-99	-7	R Occipital Pole
5.65	4.28	82	38	52	26	R Middle Frontal Gyrus
4.41	3.61		30	47	16	
5.90	4.40	68	33	27	1	R Anterior Insula
5.23	4.07		40	19	1	
4.20	3.49	55	-38	-34	-17	L Fusiform Gyrus
5.66	4.28		-38	-46	-15	
7.51	5.06	42	-30	-64	-27	L Cerebellum Ext
3.68	3.16		-20	-69	-30	
7.46	5.04	41	-18	14	3	L Caudate
5.30	4.10		-15	4	8	

Table S3. *Main Experiment – Contrast 'Reading words aloud minus Baseline'* for the control group. Statistical results of whole brain univariate analysis (T-value and Z-value were

computed at voxel-level). All peaks were significant at an uncorrected voxel-wise threshold of p < .001 and a (Family-Wise Error) corrected cluster-wise threshold with of p < .05. Regions were automatically labelled using the Neuromorphometrics atlas (http://neuromorphometrics.com/). Montreal Neurological Institute coordinates (MNI coords) were listed in millimeters in the left-right (X), posterior-anterior (Y), and inferior-superior (Z) dimensions. [L = left; R = right]

			MNI coords			
T-value	Z-value	Cluster size	X	Y	Z	Region label of main cluster
9.07	5.57	4090	-20	-59	-25	L Cerebellum Ext
8.95	5.54		33	-54	-30	R Cerebellum Ext
8.89	5.52	4090	15	-59	-22	
13.76	6.67	3924	-55	-26	1	L Superior Temporal Gyrus
11.53	6.22		-60	-4	18	L Precentral Gyrus
10.25	5.91		-50	-6	28	
11.73	6.26	3272	63	-11	6	R Superior Temporal Gyrus
10.73	6.03		65	-21	1	
10.58	5.99		53	-6	26	R Postcentral Gyrus
6.67	4.73	386	-3	-59	6	L Precuneus
5.60	4.25		10	-64	8	R Calcarine Cortex
5.46	4.18		-15	-61	8	L Precuneus
7.93	5.21	215	-13	-96	-7	L Occipital Pole
6.23	4.54		-15	-91	-17	L Occipital Fusiform Gyrus
5.24	4.07		-38	-89	-12	L Inferior Occipital Gyrus
7.14	4.92	160	-30	-61	38	L Angular Gyrus
6.74	4.76		-40	-41	36	L Supramarginal Gyrus
5.92	4.41		-33	-49	36	L Superior Parietal Lobule
6.68	4.73	132	3	-1	63	R Supplementary Motor Cortex
5.27	4.09		5	7	68	
5.69	4.30	96	15	-84	-17	R Lingual Gyrus
5.05	3.98		13	-91	-5	
3.85	3.27	96	3	-76	-7	R Lingual Gyrus
6.13	4.50	76	20	-26	58	R Precentral Gyrus
4.29	3.54		28	-29	73	R Postcentral Gyrus
6.03	4.45	74	-18	-29	66	L Postcentral Gyrus
5.21	4.06	53	0	19	23	Middle Cingulate Gyrus
3.92	3.32	53	0	12	31	

Table S4. *Main Experiment – Contrast 'Reading words aloud minus Baseline'* for the dyslexic group. Statistical results of whole brain univariate analysis (T-value and Z-value were computed at voxel-level). All peaks were significant at an uncorrected voxel-wise threshold of

p < .001 and a (Family-Wise Error) corrected cluster-wise threshold with of p < .05. Regions were automatically labelled using the Neuromorphometrics atlas (http://neuromorphometrics.com/). Montreal Neurological Institute coordinates (MNI coords) were listed in millimeters in the left-right (X), posterior-anterior (Y), and inferior-superior (Z) dimensions. [L = left; R = right]

			MNI coords			
T-value	Z-value	Cluster size	X	Y	Z	Region label of main cluster
15.81	7.02	3658	-48	-9	28	L Precentral Gyrus
11.99	6.32		-28	-14	-2	L Putamen
10.79	6.04		-63	-6	21	L Postcentral Gyrus
16.23	7.09	3168	45	-11	33	R Precentral Gyrus
15.85	7.03		53	-6	28	R Postcentral Gyrus
12.24	6.37	3168	55	-9	18	R Central Operculum
11.82	6.28	812	-15	-61	-20	L Cerebellum Ext
10.25	5.91		18	-59	-22	R Cerebellum Ext
5.99	4.44		13	-66	-45	
6.14	4.50	289	8	-69	11	R Calcarine Cortex
5.29	4.10		13	-51	3	R Lingual Gyrus
5.26	4.08		-3	-71	13	L Calcarine Cortex
5.57	4.24	102	25	-1	-27	R Entorhinal Area
4.49	3.66		33	4	-40	R Temporal Pole
7.23	4.95	63	-3	2	61	L Supplementary Motor Cortex
4.06	3.40		5	9	63	R Supplementary Motor Cortex
5.40	4.15	60	-8	14	33	R Middle Cingulate
4.51	3.67		3	19	28	L Middle Cingulate

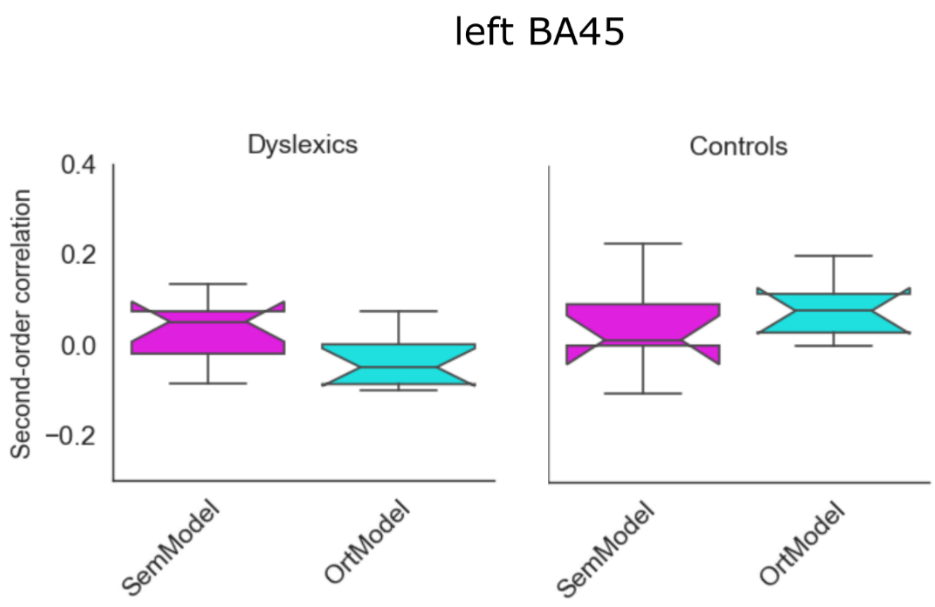

Figure S1. Second-order correlations in left BA45 for the model (semantic and orthographic) dimension for both groups (dyslexics and controls).
